# Evidence into practice: evaluating a child-centred intervention for diabetes medicine management The EPIC Project

**DOI:** 10.1186/1471-2431-10-70

**Published:** 2010-09-27

**Authors:** Jane P Noyes, Anne Williams, Davina Allen, Peter Brocklehurst, Cynthia Carter, John W Gregory, Carol Jackson, Mary Lewis, Lesley Lowes, Ian T Russell, Joanne Rycroft-Malone, Janice Sharp, Mark Samuels, Rhiannon Tudor Edwards, Rhiannon Whitaker

**Affiliations:** 1 Centre for Health-Related Research, Bangor University, Bangor UK; 2 School of Nursing and Midwifery Studies, Cardiff University, Cardiff UK; 3 National Perinatal Epidemiology Unit, University of Oxford, Oxford UK; 4 Cardiff School of Journalism, Media and Cultural Studies, Cardiff University, Cardiff UK; 5 Department of Child Health, Wales School of Medicine, Cardiff University, Cardiff UK; 6 Pharmacy Department, Royal United Hospital Bath, Bath, UK; 7 Centre for Adolescent and Child Health, University of Western England, Bristol UK; 8 School of Medicine, Swansea University, Swansea UK; 9 Media Resources Centre, University Hospital of Wales, Cardiff, UK; 10 Formerly, Roche Diagnostics (UK) Ltd, now Head, Office for Clinical Research Infrastructure (NOCRI), London UK; 11 Centre for Economics and Policy in Health, Bangor University, Bangor UK; 12 North Wales Organisation for Randomised Trials in Health (NWORTH), Bangor University, Bangor UK

## Abstract

**Background:**

There is a lack of high quality, child-centred and effective health information to support development of self-care practices and expertise in children with acute and long-term conditions. In type 1 diabetes, clinical guidelines indicate that high-quality, child-centred information underpins achievement of optimal glycaemic control with the aim of minimising acute readmissions and reducing the risk of complications in later life. This paper describes the development of a range of child-centred diabetes information resources and outlines the study design and protocol for a randomized controlled trial to evaluate the information resources in routine practice. The aim of the diabetes information intervention is to improve children and young people's quality of life by increasing self-efficacy in managing their type 1 diabetes.

**Methods/Design:**

We used published evidence, undertook qualitative research and consulted with children, young people and key stakeholders to design and produce a range of child-centred, age-appropriate children's diabetes diaries, carbohydrate recording sheets, and assembled child-centred, age-appropriate diabetes information packs containing published information in a folder that can be personalized by children and young people with pens and stickers. Resources have been designed for children/young people 6-10; 11-15; and 16-18 years.

To evaluate the information resources, we designed a pragmatic randomized controlled trial to assess the effectiveness, cost effectiveness, and implementation in routine practice of individually tailored, age-appropriate diabetes diaries and information packs for children and young people age 6-18years, compared with currently available standard practice.

Children and young people will be stratified by gender, length of time since diagnosis (< 2years and > 2years) and age (6-10; 11-15; and 16-18 years). The following data will be collected at baseline, 3 and 6 months: PedsQL (generic, diabetes and parent versions), and EQ-5 D (parent and child); NHS resource use and process data (questionnaire and interview). Baseline and subsequent HbA1c measurements, blood glucose meter use, readings and insulin dose will be taken from routine test results and hand-held records when attending routine 3-4 monthly clinic visits.

The primary outcome measure is diabetes self-efficacy and quality-of-life (Diabetes PedsQL). Secondary outcomes include: HbA1c, generic quality of life, routinely collected NHS/child-held data, costs, service use, acceptability and utility.

**Trial Registration:**

ISRCTN17551624.

## Background

### The requirement for health information

People of all ages require high quality information promoting health, self-care and medicines management to help facilitate their engagement in participative models of health care, and assist them in making choices [1-4]. In the United Kingdom (UK) the National Health Service (NHS) Constitution makes clear that patients require information to engage fully and knowledgably in decision-making, and be aware of risks and benefits of treatment options [4].

Policy makers also identify a need for health and social services providers to increase capacity, confidence and efficacy of individuals for self-care and to build social capital in the community [4-10]. The requirement for prevention, early intervention and support for individuals for self-care, and promoting wellbeing for the wider population is a fundamental policy aspiration [4-10]. However, there is uncertainty about the positioning of children, young people and their families within these models and policies and what practical plans and processes exist for successful implementation.

Children's age-appropriate and child-centred health information is likely to be critical to developing self-care and wellbeing as children's autonomy increases with age [9]. Information needs and informed choice are central to the Children's National Service Framework (NSF), (including a standard on medicines management, and the Children's Plan, which make specific reference to the requirement to provide high-quality, age-appropriate, child-centred information in varying formats) [9-11]. There is however little reliable evidence concerning the effectiveness of different types of provision of health information for children and young people. There is even less evidence about types and formats of information which could empower children and young people to make decisions and choices, where appropriate, about aspects of their care [12].

Progress has been made on a UK strategy for service delivery and organisation of medicines for children and young people to facilitate not only a measurable increase in appropriately labelled and formulated medicines and conduct of trials, but also information for prescribers, carers and children [9]. One outcome is the setting-up of the Medicines for Children Research Network (MCRN), [13] which is supporting the EPIC project and linked foundation study the Information Matters Project (IMP), funded by the National Institute for Health Research: Service Delivery and Organisation [14,15].

### Broad policy background

The need for child-centred, age-appropriate information on medicines specifically, and self-care management in general, is highlighted when viewed against the broader NHS public health policy context. Children's health policy is centred on the notion of 'family-centred' care with family members providing a large proportion of care, and with children taking on more responsibility for their healthcare as they gain autonomy. The Children's NSF model of children's acute and chronic disease management focuses on educating children/young people in age-appropriate ways to deliver aspects of their own healthcare, and specifically identifies parents as experts [4]. The shift in focus to homecare and community settings requires complex arrangements for medicines and treatments and greater support for parents and children/young people who are administering increasingly complex medicines (eg insulin pumps), and treatment regimes, and who are recommended to adapt their lifestyles to optimise health [9]. Information on self-administration and medicines management is required to support delivery of children's healthcare in various community settings (eg home and school) [9].

The illness trajectories of many childhood conditions now extend into adulthood [16]. There is little information available for young people and their families around transition between child and adult service provision, with many young people seemingly unprepared to manage their own care and live independently [17]. Findings from an overlapping SDO study looking at transition of young people to adult services (TCADS) are expected soon [18]. Available standard patient information is often of poor quality and may not be easily accessible or understandable for children, young people and their families [1].

Policies need to be placed within the context of children and young peoples' lives, illnesses they experience and what best suits their needs. Long-term conditions such as diabetes are commonly treated with medicines and children/young people increasingly take responsibility for their regimes over time, especially during school hours [19]. Children and young people need to be involved with their families/carers and professionals in decision-making about their care-management, including understanding risks and benefits, and specific instructions to ensure optimum effect [9]. Research has been aimed at identifying aspects of structured education programmes, for example comparing their effectiveness, [20] developing innovative curricula, [21] and exploring acceptability to adolescents and their parents and eliciting ideas on how they would set about designing education sessions [22]. There is also work on psycho-educational interventions [23].

Clinical guidelines indicate that high-quality, child-centred information underpins the achievement of optimal glycaemic control with the aim of minimising acute re-admissions and reducing the risk of complications in later life [24]. There is, however, insufficient evidence about the effectiveness of information underpinning diabetes education and medicines management for children and young people [25-27]. Likewise tailored, child-centred information could equip children and young people with the knowledge to become expert in diabetes care [23,24].

Building on research evidence determining children and young people's preferred types and formats of information across five tracer groups and associated critical discourse analysis derived from the linked IMP qualitative project, the pragmatic trial protocol reported here aims to fulfil a significant gap in current knowledge concerning children's diabetes information and its use in routine practice to promote optimal self-care and medicines management [28].

## Methods/Design

### Research aims

The aim of the research is to develop and evaluate an individually-tailored, age-appropriate diabetes diary and information pack to support decision-making and self-care relating to insulin management and electronic blood glucose monitoring for children and young people aged 6-18 yrs with type 1 diabetes, compared with available resources in routine clinical practice.

### Objectives

To:

1. Review gold-standard clinical guidelines, currently available information including findings from the linked qualitative IMP study to identify best practice, and types/formats of information most likely to assist age-appropriate decision-making and choices concerning blood glucose monitoring and insulin management.

2. Develop an age-appropriate information intervention (child-centred diabetes diary and information pack) for children and young people, to support appropriate use of blood glucose meters to optimise management of and concordance with their insulin regime.

3. Explore the utility of the child-centred diabetes diary and information pack (in this context utility refers to ease of use and fitness for purpose) within different contexts in which children and young people manage their routine diabetes care (home, school, community) with and without support from parents or healthcare professionals, and in alternative settings.

4. Explore how children/young people with and without their parents, teachers, nurses, doctors use (or not) the diabetes diary and information pack to support decision-making; in particular how children and parents 'self-prescribe' the correct (or incorrect) dose of insulin.

5. Identify similarities and differences between the diabetes diary and information pack developed for adolescents and those available within adult diabetes services.

6. Evaluate the diabetes diaries and information pack within the context of routine diabetes care in relation to patient outcomes (diabetes-specific health-related quality of life, generic health-related quality-of-life, medicine and treatment concordance, acceptability, ease of use, and glycaemic control).

7. Identify gaps in knowledge to inform a future research agenda.

### Design

To meet our objectives, which are aligned with the phases of the MRC Framework for RCTs of complex interventions, a four-stage study has been designed [29,30].

Stages 1 and 2 to develop the information intervention have mostly been completed and will be described briefly. This protocol focuses on Stage 3, the randomised controlled trial to evaluate the effectiveness and cost effectiveness of the children and young people's age appropriate diabetes diaries and information packs developed in stages 1 and 2.

The four stages are as follows:

Stage 1. Review and, where appropriate, undertake further work to identify types/formats of information most likely to assist age-appropriate decision-making/choices related to children/young people with type 1 diabetes.

Stage 2. Construct age appropriate diabetes diaries and information packs, and consult with children as appropriate.

Stage 3. Conduct a pragmatic evaluation to assess utility, acceptability, effectiveness and cost effectiveness of the diabetes diaries and information pack.

Stage 4. Undertake data synthesis and comparative analysis of stages 1-3.

### Conceptual and methodological frameworks

In addition to using the MRC Framework for designing and evaluating complex interventions, the Promoting Action on Research Implementation in Health Services (PARIHS) framework will be used as the framework for the translation of evidence into practice evaluation [31,32]. The framework has been theoretically and empirically developed to represent the interplay and interdependence of the many factors influencing implementation of evidence into practice. This is explained by a function of the relation between evidence, context and facilitation [32-34]. The hypothesis offered is that for implementation of evidence to be successful there needs to be clarity about the nature of the evidence being used, the quality of context, and, the type of facilitation needed to ensure a successful process. The framework has been used by others to inform the design and evaluation of evidence into practice initiatives [35-37]. PARIHS framework is particularly relevant to this study because:

1. It aims to introduce new diabetes diaries and assembled information packs (evidence) into children's self-care regime and healthcare practice in order to improve blood glucose meter use and insulin management. Understanding the factors that influence its implementation and use will be important in determining the acceptability and feasibility of the information pack (facilitation)-this framework will provide a conceptual guide for mapping these issues.

2. Understanding how the information pack is used in different contexts where children/young people manage their diabetes will be key in the evaluation of its utility and contribution. Applying the framework will allow a focus on the key contextual variables mediating the implementation and use of the information pack.

3. It facilitates the gathering of individual (e.g. child/practitioner/carer) experiences as well as appreciating the fit with the broader context of care delivery.

## Plan of Investigation

### Stage 1. Review of literature and discourse analysis of currently available children's health information

#### Literature review

We are undertaking an ongoing systematic review of literature, policy, evidence of cost-effectiveness, best practice clinical guidance and management plans that will run throughout the study. We have used a scoping review of current evidence from the IMP project to inform intervention development and have extended the focus on childhood diabetes diary and information pack development.

#### Critical discourse analysis of currently available childhood-diabetes information sources

We have used completed IMP project critical discourse analysis findings to inform intervention development and have focused in detail on childhood diabetes. We have explored management of childhood diabetes and focused on blood glucose monitoring and insulin management as a key exemplar concerning medicine management, self-care and concordance. We have also looked specifically for similarities and differences in the discourses and philosophies underpinning children's/young peoples' and adult care pathways and management plans to see how and in what ways medicine management and self-care discourses/philosophies change at key stages across the lifespan. Information sources across all mediums and sectors (eg. NHS, pharmaceutical) were sought. This work established what sources of diabetes information were currently available to children/young people and their families. We have also identified the underlying assumptions of the information sources and their main messages, and we have assessed their applicability in terms of age, disability, ethnicity and gender, and for those children living away from their families. Analysis of the content has identified whether key messages match clinical guidance on childhood diabetes management.

### Stage 2. Diabetes diaries and information pack development

#### Expert clinical advisory group

Towards the end of phase 1, we convened an expert clinical group to discuss what gaps existed in current children's diabetes information, and what new information could be produced within the study budget and time constraints. It was decided that evidence concerning preferred formats and presentation of age-appropriate key health messages would be used to design and develop a series of age-appropriate children's diabetes diaries that record daily insulin management and blood glucose measurements, and carbohydrate counting sheets. In addition, the diary would be central to an age-appropriate information pack of existing diabetes and diabetes-related information resources that were considered high quality in terms of content and identified by children and young people in focus groups and interviews as being attractive and relevant to them in terms of content and presentation.

Evidence from stage 1 (ongoing literature review and discourse analysis) has been used as an empirical basis for developing the age appropriate diabetes diaries and information packs. The diabetes diaries and information pack were designed in conjunction with children/young people, parents, healthcare professionals, and a children's medical illustrator.

#### Qualitative interviews and focus groups

To establish the context for the development of the diabetes diaries and information packs and their implementation in routine practice, linked with the IMP project, we have conducted 7 focus groups and 48 interviews to ascertain children and young people's views of currently available information resources across 5 tracer conditions including diabetes, and explored children and young people's information needs related to managing their condition and self-care, including diabetes. We also interviewed 52 parents and 11 healthcare professionals and undertook non-participant observations of routine clinical encounters where information is exchanged.

Over the course of the EPIC study, we plan to increase the number of interviews with children and young people with type 1 diabetes to around 20, with the specific aim of including those who live or have lived away from their families in the short, medium or long term (such as summer camp, school trips, foster or institutional care etc) to ascertain children and young people's views and experiences of managing their diabetes in various everyday contexts.

In addition, approximately 20 healthcare professionals drawn from participating trial sites (approximately two from each trial site), will be interviewed to document current routine practice and local clinical care pathways for children with type 1 diabetes in their sites.

#### Obtaining children and young people's perspectives on various iterations of the age appropriate diabetes diaries and information packs

With permission from the organisers of family days run by children's diabetes charities and support groups, we have asked for children and young people's general views on various iterations of the age appropriate diabetes diaries and information packs. They have commented on artwork, colours, formats, sizes and types of information. We have also used advertisements in charity and Roche family support network news letters and communications inviting children and young people to visit the EPIC Project website. Having given their online consent (and parent/guardian consent if under 16 years of age) children and young people were invited to take part in web-based activities such as choosing which image they liked best out of a selection, and commenting on various iterations of the information resources in production.

#### Children's diabetes information resources produced for the trial

In an iterative approach, integrating findings from the linked IMP Project, and building throughout stages 1 and 2, we have produced the following range of resources that can be individually tailored for pragmatic evaluation in routine clinical practice-stage 3:

• Three diabetes diaries for children and young people using multiple insulin injections (6-10 years, 11-15 years and 16-18 years);

• One diabetes diary (6-18 years) for children and young people using insulin pumps;

• Child-friendly sheets for recording carbohydrate intake;

• Three age-appropriate information packs containing published diabetes information bound in an age-appropriate folder (6-10 years, 11-15 years and 16-18 years), and

• Stickers and pens for children and young people to personalise their folder.

### Stage 3. Trial platform to evaluate the diabetes diaries and information packs in routine practice

#### Methods

##### Study design

**Figure 1:**
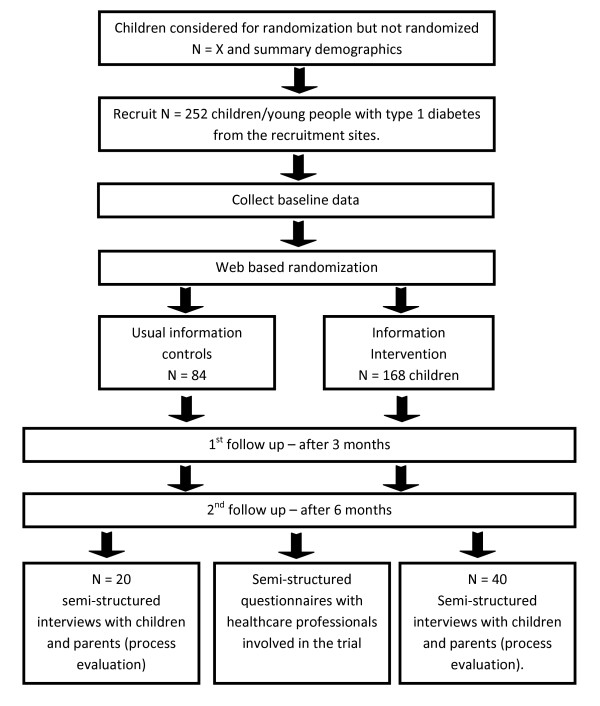
**Flow of patients through the trial**.

This trial is an individually randomised controlled trial with two parallel groups. The protocol is summarised in Figure 1.

##### Main hypothesis

Children and young people with type 1 diabetes aged from 6-18 years, receiving and using an individually-tailored and age appropriate diabetes diary and information pack will increase their diabetes-related self-efficacy and quality of life at 6 months.

##### Secondary hypotheses

That receiving and using an individually-tailored and age-appropriate diabetes diary and information pack will lead to an improvement in HbA1c, generic health-related quality of life, and a range of care process outcomes, and be cost-effective.

##### Setting

NHS children's, transition, and young adult diabetes clinics, and a range of domestic/community settings (eg family homes and schools).

##### Site selection and preparation

Depending on the size of site and number of children/young people with diabetes type 1, we envisage up to 10 sites (depending on current NHS re-organisation and amalgamation of Trusts) will recruit children and young people. We will be guided by co-applicant clinicians and the MCRN/CRC Cymru research network who have an overall view of available of sites for trials and will have an overall strategic role in supporting research teams to facilitate site and participant recruitment [38].

The age-appropriate diabetes diaries and information pack will be individually-tailored and introduced by nurses/doctors in children and young people's diabetes clinics during routine visits. We will hold a launch event to familiarise healthcare professionals with the information pack in each participating site.

### Types of participants

#### Inclusion criteria

Children age 6-18 years with type 1 diabetes.

#### Exclusion Criteria

Children/young people with:

1. Needle phobia;

2. Any significant social or emotional problems where such problems in the opinion of the clinical team are likely to impair a child's ability to take part in the trial;

3. Any significant physical or intellectual impairment which in the opinion of the clinical team is likely to impair a child's ability to take part in the trial, and

4. An inability to communicate in an age appropriate way in written and spoken English.

The underlying principle guiding clinicians is that children/young people should be entered into the trial where at all possible and should only be excluded if being in the trial would be detrimental to their social, emotional or physical health, or children/young people or their parents are unwilling to give their informed consent/assent.

#### Sample size calculation and effect size

A systematic review provides sample size calculations for studies of educational interventions targeting psychological effects and glycaemic control (HbA1c) for children with diabetes [39]. They calculated a total of 130 randomized subjects in order to detect a 0.5 (medium) psychological effect size, with a power of 80% at the 0.05 significance level (assuming equal assignment in the two arms). They report that the effect size for psychological outcomes is more predictable with a median and mean of 0.38 and 0.35 respectively, therefore we will aim to detect an effect size of 0.4.

The sample size is 252 children/young people with type 1 diabetes (this is allowing for a 10% drop out rate, and adjusting for a 2:1 randomisation strategy). In order to gain more experience of the new intervention in routine practice, we will employ a 2:1 randomization strategy and randomise 168 children/young people into the intervention arm and 84 children/young people into the non intervention arm, stratified by age, gender and length of time since diagnosis (< 2 years and > 2years). Figure 1 shows the flow of patients through the trial.

##### Recruitment

Where appropriate consultants, nurses, or MCRN/CRC Cymru research nurses will send an information pack to a child/young person (and if under 16 years their parent/guardian) attending a clinic one week prior to the clinical appointment. During the clinic visit, the consultant/nurse/research nurse will ask them if they want to take part in the study. If the child/young person agrees to take part in the study, the consultant/nurse/research nurse will take the consent from young people over 16 years, assent and parent/guardian proxy consent from children under 16 years. Children and young people not entered into the trial will receive standard care.

##### Allocation

Children age 6-18 years fulfilling the inclusion criteria and for whom appropriate consent(s) (proxy if appropriate) are obtained will be randomized using an independent web based randomisation service supported by the North Wales Organisation for Randomised Controlled Trials in Health and Social Care (NWORTH).

##### Randomisation

A web-based, password protected, secure adaptive randomisation with a telephone backup has been developed.

168 children/young people will be randomized into the intervention arm and 84 children/young people into the no intervention arm, stratified by gender, length of time since diagnosis (< 2 years and > 2 years) and age (stratification by age will be into the following age categories: 6-10 years; 11-15 years; and 16-18 years).

#### Planned interventions

##### Group 1 - Information intervention

An age-appropriate diabetes diary and information pack produced or assembled for the study will be selected and individually-tailored as appropriate to each child/young person depending on their age and diabetes management, and introduced by nurses/doctors and as appropriate other multi-disciplinary team members in clinic during routine visits by children/young people between the ages of 6-18 years with type 1 diabetes. Parents of children under 16 years will be provided with verbal and written guidance on supporting their child's use of the information pack. Parents attending clinic consultations with young people over 16 years will receive the same verbal advice as the young person, and written guidance will be aimed at the young person.

##### Group 2-Standard practice

Children age 6-18 years with type 1 diabetes receiving standard practice will be the 'practice as usual' control group. A manual of standard practice for each centre will be produced. This will help with the comparisons of outcomes at the end of the trial.

#### Outcome measures

##### Primary outcome measures

Choice of outcomes is guided by a Health Technology Assessment commissioned systematic review recommending that HbA1c (glycaemic control measure) is not the appropriate primary outcome on which to assess benefits of an intervention designed to more directly effect behaviour/self-management [39]. Therefore, the primary outcome measure is diabetes self-efficacy and quality-of-life as measured by the single composite score derived from the Diabetes PedsQL.

##### Secondary outcome measures

Secondary outcomes include: HbA1c, generic quality of life, routinely collected NHS/child-held data, costs, service use, acceptability/utility.

#### Baseline, 3 and 6 month data collection

Children/young people (if appropriate with support of, or proxy report by parents) will complete a baseline questionnaire recording sociodemographic variables, patient characteristics, and PedsQL (generic, diabetes and parent versions). The EQ-5D will be completed by parents (as a proxy measure) as well as the child/young person.

Follow-up questionnaires, containing the same outcome measures as at baseline, and with an additional focus on process will be administered at 3 months and 6 months, (including data on health service use, episodes of diabetic ketoacidosis, and all hospital admissions for acute complications). Non-responders will receive telephone/postal reminders after two and four weeks.

Baseline and subsequent HbA1c measurements and routinely collected NHS/child-held data will be collected by the clinicians/diabetes nurse specialists/MCRN nurses, or researchers where appropriate. Blood glucose meters will be checked for the previous 250 blood glucose records if considered appropriate by the clinician and if used as part of routine clinical practice.

### Implementation, service utilisation and costs

#### Economic Evaluation

Murphy et al., strongly recommend that cost-effectiveness is considered as an outcome as none of the studies in their review of psycho-educational interventions with adolescents addressed it [39]. We will therefore weigh up the diabetes related costs and consequences of the different interventions (that involve resource use) from an NHS perspective. Contacts with NHS services and resource use will be collected via questionnaire. Costs will be obtained from national sources. Activity will be collected for 6 months.

#### Process evaluation

Following the intervention, sixty children/young people and parents (as appropriate) will be interviewed by telephone or in person (40 from the intervention group and 20 from the control group) in order to gain further understanding about implementation issues, and user experiences of the intervention diabetes diaries and information packs or resources used in existing routine practice.

Healthcare professionals associated with the care of children/young people recruited to the trial will also be invited to complete a semi-structured questionnaire to determine acceptability and impact of the new diabetes diaries and information pack in practice.

#### Data handling

SPSS^® ^and Atlas Ti^® ^will be used for statistical, qualitative and healthcare professional questionnaire data handling [40,41]. NWORTH will support data management and processing. Where appropriate, we plan to use an electronic data collection system (TrialSys^® ^) in each centre with an alternative paper-based system [42]. Data will be transmitted securely electronically to the clinical trials unit NWORTH [43].

#### Data analysis

##### Statistical analysis

Initial descriptive statistics will describe characteristics and demographics of the sample at baseline. We shall compare outcomes between the two groups by analysis of covariance to adjust for possible differences in baseline measurements. This will be repeated at 3 and 6 months comparing intervention and control groups. In addition, longitudinal analysis will consider any changes over time. These analyses will examine changes in the quality of life measures (paediatric EQ-5D, PedsQL generic and diabetes-specific health measures) over baseline, both using a pairwise comparison, studying change on individuals, and a cohort analysis comparing overall change in group means.

Multiple regression analyses will be performed to identify factors which predict good outcomes within and between groups.

#### Cost effectiveness analysis

We will undertake a cost-utility analysis, whereby costs are in monetary terms and outcomes are in preference-based non-monetary units such as Quality Adjusted Life Years (QALYs). The area under the curve method will be used for calculating QALYs weighting survival by quality of life weights measured using the paediatric EQ-5D instrument. We will compare our findings with the unofficial NICE ceiling of £30,000 per QALY. Discounting will not be necessary given the time period.

#### Uncertainty

The bootstrap calculation is a useful statistical approach for examining the uncertainty in cost-effectiveness analysis. It is a non-parametric simulation method used when the underlying data has a skewed distribution. The bootstrap method can be used to provide an estimate of the probability distribution of the cost-effectiveness ratio, its confidence interval, or variance in the ratio.

#### Qualitative data analysis

Interviews will be tape recorded and transcribed. The process analysis accompanying the subsequent evaluation will explore implementation issues and compare the experience of managing diabetes and insulin management and self-care processes between the intervention and control pathways. The predominantly deductive 'framework approach' will be used to categorise qualitative data based on the literature, conceptual framework, the trial design, and the evaluation focus [44].

#### Healthcare professional questionnaires

Healthcare professionals' questionnaire data will be analysed using descriptive statistics and open ended questions will be subject to content analysis.

### Stage 4. Data synthesis and comparative analysis

Data from stages 1 to 3 will be synthesised and subject to comparative analysis.

#### Ethical arrangements

##### Ethical approval

A favourable opinion has been received from Cardiff Research Ethics Committee. Reference **08/MRE09/57**

##### Risks and anticipated benefits for trial participants

The research carries minimal risk and therefore it is considered appropriate to involve children and young people in evaluating the diabetes diaries and information packs [45].

**Figure 2:**
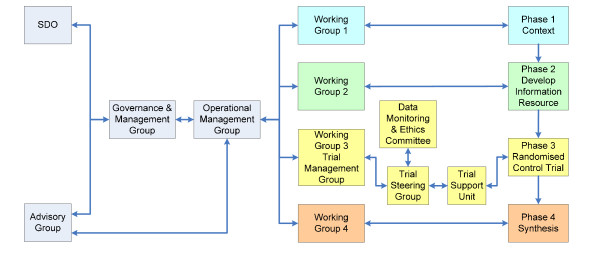
**Trial governance and management arrangements**.

We have convened an expert clinical advisory group and developed a specific clinical governance and risk management framework with diabetes clinicians to quality assure and 'sign off' children's diabetes information produced for the study as appropriate for use in the NHS (see Figure 2).

Team members with direct contact with children will undergo appropriate Child Safeguarding screening, and follow local child safeguarding procedures in each site.

##### Consent for participation in the trial

Participants will be children/young people between the ages of 6 to 18 years. Young people over the age of 16 can provide their own consent, however consent by proxy (from a parent or guardian) will be obtained for children under the age of 16 years. Assent will be sought from children under 16 years.

## Discussion

The multi-disciplinary team and innovative design will ensure that children's needs are made explicit within policy implementation and service delivery, providing generalisable evidence to: 1. Identify types/formats of information to underpin structured education programmes for children with type 1 diabetes. 2. Guide integration of high quality children's information needs within service delivery to facilitate choice in routine clinical practice at national and local levels. 3. Inform decisions about the allocation of NHS resources in relation to service use. 4. Assist the development of a future research agenda related to age appropriate, child-centred information across a range of conditions, in order to fill an identified service delivery and organisation gap.

We have also successfully integrated high levels of participation with children, young people, families and healthcare professionals in the study at various key stages. The study will undoubtedly also yield further useful and valuable evidence on process and outcome measurement generally in a range of children and young people that can be used to inform the design of future trials of information and behavioural interventions generally across a range of tracer conditions. Evidence that addresses the preparation of children in order to manage their own care and live independently into adulthood is critical to the NHS agenda

The study complements other children's diabetes studies undertaken by team members and findings will contribute to a developing a wider evidence base with specific reference to children/young people with type 1 diabetes [14,18,46].

We have been asked by NIHR SDO to publish the trial management and research governance framework developed for EPIC.

### Trial management and research governance framework

#### Team management

Drawing on team members' experiences of managing teams, we have adapted a template shared by PB and constructed a management and research governance framework to support the four phases of our study (1. context: review literature and current information 2. develop information pack, 3. randomized controlled trial, 4. synthesis of phases 1-3 and comparative analysis), and to ensure cohesive and effective working between team members (see Figure 2).

#### Key Groups

Group tasked with overall responsibility and governance: 'Governance and Management Group'

Management and research governance is being overseen by a group composed of the co-applicants named on the bid, led by AW and JN. Co-applicants were chosen to support this application on the basis that they each have established reputations in fields and expertise most relevant to the proposed research, including: systematic review skills, research skills, trials expertise, experience of user groups/working with users, clinical expertise, policy knowledge, services planning and development, management and delivery, multi-agency/disciplinary partnerships, networking and dissemination skills. In addition, co-applicants have specialist roles within relevant clinical research thematic networks (PB, JG, JN) and professional bodies (JG, CJ, ML, JN, AW).

All co-applicants are experienced and effective communicators in their capacity as researchers, clinicians and managers. The majority of us have collaborated previously or are currently collaborating on other projects. While we each bring different discipline and professional perspectives to bear on the objectives we have set, we aim to promote team values with an inclusive approach where differing opinions are respected. Many of us have developed considerable confidence as team leaders in multi-disciplinary/multi-agency settings. The Governance and Management Group will normally meet once a year, and will report to the funder. Additionally, teleconference meetings will be called if required.

Group tasked with day-to-day management: Operational Management Group

Day-to-day management of operational aspects of the programme will be the responsibility of the two PIs working with the two RAs employed for the duration of the study. AW will work with one RA based in Cardiff, meeting face-to-face on a weekly basis and JN with one RA based in Bangor, also meeting face-to-face weekly. This core group of four researchers will be in close contact with each other via a secure web site and will communicate via teleconference or telephone at least every 2 weeks during the 36 months of the programme. They will meet face to face in the early stages to establish working relationships. Our experience suggests this increases effectiveness and efficiency. They will also be in contact with members of the Working Groups, Governance and Management Group and Advisory Group as detailed in the section 'Working together effectively' below. Management of information will be critical to the day-to-day management of the study; common data bases and resources have been set up and it has been important to identify precise division of labour from outset of study.

#### Working together effectively

Based on previous experience and current models of good practice employed on projects by the co-applicants, we have set up Working Groups. These will give clarity to the roles and responsibilities of the co-applicants who have been costed into the price of the proposed research. They are planned to provide particular advice and guidance to the PIs and RAs in each phase of the study: 1. Context: review of literature and current information 2. Development of the information pack, 3. Randomized controlled trial, 4. Synthesis of phases 1-3 and comparative analysis. Guidance will be related to varying aspects such as range of policy, best clinical practice, child/user-friendly construction of question sheets and so on. Guidance on dissemination of findings will be a key aspect of their role. Individual members of the Governance and Management Group are leading activity in the working groups, working closely with the Operational Management Group. Advisory Group members will be called on for specific expertise, including dissemination skills. Each working group reports to the Governance and Management Group via the Operational Management Group.

#### Trial Management

The North Wales Organisation for Randomized Trials in Health and Social Care (NWORTH) will support implementation and running of the trial.

Working group three in Figure 2 has been convened as a Trial Management Group, Chaired by LL (co-applicant) that meets monthly by teleconference. Membership includes PIs, ROs, trial statistician, and health economists.

#### Trial Steering Group

A trial steering group (TSG) has been convened under the independent Chairmanship of Professor Tim Barrett. The TSG includes clinical, academic, parent and young person representation and will meet every six months in person and every six months by telephone conference.

#### Data Monitoring and Ethics Committee

An independent data monitoring and ethics committee (DMEC) has been convened and chaired by Dr Chris Foy (independent statistician), who will report to Professor Tim Barrett (Chair TSG). The DMEC will meet face to face every six months and virtually every six months prior to the TSG meeting.

#### Serious Adverse Event Reporting

A serious adverse event monitoring and reporting system has been devised in conjunction with NWORTH.

#### MCRN and CRC Cymru

The study has been adopted and supported locally by MCRN, Diabetes Research Network, and Children and Young Peoples' Network CRC Cymru.

#### Additional Stakeholder and lay input

In addition to the clinical expert advisory group described previously, we wish to involve at various stages of the study those people we have already consulted in preparing the research proposal-parents, children, young people-and others such as clinicians, managers and voluntary sector representatives. A number are working with us on the linked IMP project.

Additional stakeholder and lay advice and input will therefore be drawn from people with knowledge and experience relevant to the aims of the project. Previous experience of this type of research by the co-applicants suggests that collaboration, communication and transparency with key stakeholders from the outset will be crucial to the success of the proposed study.

## Competing interests

MS is a former employee of Roche Diagnostics.

## Author's contributions

JN and AW are chief investigators and were involved in the conception of the study. All co-applicants contributed to the design of the study and/or development of the protocol. Artist JS produced illustrations and artwork for the intervention. JN and AW drafted the manuscript with all authors providing critical review and final approval. JN is involved with supervision of students on this project.

## Authors' information

AW and JN (joint-PIs) have track-records in managing research programmes in patient/carer-centred service delivery and organisation.

JN has experience in child health research, health services research and health economics and evidence synthesis.

AW has a specific interest in policy changes in relation to the shaping of new and changing professional roles, identities and relationships, and changing patient engagement with the health services.

PB, as Chair of the Methodology Clinical Studies Group of MCRN will act as formal link between the research team and MCRN and facilitate additional links with the MCRN Endocrinology Clinical Studies Group. As an experienced trial researcher, PB will provide additional input and scrutiny into the running of the trial and data monitoring.

The study demands applied clinical expertise and research. JG (Paediatric Endocrinology) and LL (Children's Diabetes Specialist Nurse) have a joint-research programme into delivering children's diabetes care.

As an academic clinician managing a caseload of children with diabetes, JG will help facilitate recruitment and support the development of a robust risk management framework for the use of the information pack in practice. As an experienced trial researcher, JG will provide additional input and scrutiny into the running of the trial and data monitoring. He will also provide cross-linking with another trial on which he is principal investigator into adolescent diabetes care, thereby adding value by sharing best practice and findings. JG through his membership of British Society of Paediatric Endocrinology has strong links with the MCRN Endocrinology Clinical Studies Group and will facilitate communication on behalf of the study. He also has links with the Diabetes Research Network.

As diabetes nurse specialist and academic, LL will provide advice on clinical governance issues and support the development of a robust risk management framework for the use of the information pack in practice.

CJ is a children's pharmacist and will advise on current policy and practice in relation to administration of children's medicines and the children's British National Formulary. She will advise and support on the development of appropriate risk management procedures with specific reference to medicines, and support the facilitation of translation of the information pack into practice with reference to pharmacy professionals.

ITR is non executive director of the North Wales Clinical Trials Unit which will coordinate trial management and scrutiny. ITR will also oversee statistical aspects. He is also Professor of Clinical Trials, Centre for Health Information Research & Evaluation, Swansea University School of Medicine.

RW is trials unit assistant director (NWORTH) and research statistician. The clinical trials unit will develop randomisation procedures and provide an independent randomisation service, advise on data base development, data storage, and provide data as requested by the data monitoring and ethics committee.

DA has expertise in policy and research concerning transition from child to adult. Her extensive work on policy analysis from a sociological perspective and her depth understanding of theories informing current policy supports her role in advising on these matters in relation to children and young people with type 1 diabetes.

CC (specialist in children, communication and media) will advise on the development of the information pack in terms of content and intellectual structure from a media perspective.

As a member of the Wales Health Economics Group, RTE brings key expertise to the study. She will have overall responsibility for the health economics component of the trial.

Translating evidence into practice is critical to the research plan. J R-M has extensive experience in knowledge translation and utilisation, and will guide translation of theoretical concepts and findings from linked studies to practical application and evaluation in the current study.

As a children's researcher, lead nurse and clinical quality lead in a paediatric clinical trial, ML will provide advice on clinical governance issues and support the development of a robust risk management framework for the use of the information pack in routine practice.

MS will help facilitate publication of EPIC advertisements in Roche newsletters to children and families who have signed up to receive information. He will also provide a link with industry, who produce blood glucose monitors and insulin and are required to produce user friendly product information (currently for adults only). MS will receive no financial reimbursement for supporting the study. Note MS commenced as Managing Director, NIHR Office for Clinical Research Infrastructure (NOCRI), April 2010.

JS, a children's medical illustrator, will produce illustrations and support the design of the information pack.

## Pre-publication history

The pre-publication history for this paper can be accessed here:

http://www.biomedcentral.com/1471-2431/10/70/prepub
